# Discovery of carbon nanotubes in sixth century BC potteries from Keeladi, India

**DOI:** 10.1038/s41598-020-76720-z

**Published:** 2020-11-13

**Authors:** Manivannan Kokarneswaran, Prakash Selvaraj, Thennarasan Ashokan, Suresh Perumal, Pathikumar Sellappan, Kandhasamy Durai Murugan, Sivanantham Ramalingam, Nagaboopathy Mohan, Vijayanand Chandrasekaran

**Affiliations:** 1 Science Branch, Indore Division, Archaeological Survey of India, Indore, India; 2 Department of Chemistry, School of Advanced Sciences, Vellore Institute of Technology, Vellore, India; 3 Department of Physics and Nanotechnology, SRM Institute of Science and Technology, Kattankulathur, Chennai, India; 4 Department of Mechanical and Aerospace Engineering, University of California, San Diego, CA 92093 USA; 5 Department of Bioelectronics and Biosensors, Alagappa University, Karaikudi, India; 6 Department of Archaeology, Government of Tamil Nadu, Chennai, India; 7 Nano Mission, Technology Bhavan, Department of Science and Technology, Ministry of Science and Technology, Government of India, New Delhi, 110016 India; 8 Present Address: Rolls-Royce High Temperature Composites, 5730 Katella Avenue, Cypress, CA 90630 USA

**Keywords:** Materials science, Nanoscience and technology, Chemistry

## Abstract

Unique black coatings were observed in the inner wall of pottery shreds excavated from Keeladi, Tamilnadu, India. Raman spectroscopy, transmission electron microscopy, and X-ray photoelectron spectroscopy were used to understand the nature of the coating. The analysis revealed the presence of single, multi-walled carbon nanotubes and layered sheets in the coating. The average diameter of single-walled carbon nanotube found to be about 0.6 ± 0.05 nm. This is the lowest among the single-walled carbon nanotubes reported from artefacts so far and close to the theoretically predicted value (0.4 nm). These nanomaterials were coated in the pottery’s that date backs to sixth century BC, and still retain its stability and adhesion. The findings of nano materials in the pre-historic artifacts, its significance and impact are discussed in this article.

## Introduction

Synthesis and usage of nanomaterials are not the outcome of modern development in science and technology. There are reports documented the usage of nanomaterials in the ancient arts and tools indicating that ancient days humans were aware of the uniqueness of these materials and the methods of synthesizing it. But they might not know the scientific principles at the nano scale and its unusual properties. For instance, the presence of Cu and Ag nanoparticles has been identified in glazed Islamic potteries^1^ and the Renaissance pottery from Mediterranean region^2^. These 0-D metal nanoparticles were used to improve the lustre of the potteries dates back to eighth–nineth century AD^3^. Reibold et al. observed the presence of 1-D nanomaterials such as multi-walled carbon nanotubes (MWCNT) in Damascus steel^4^. It explains the superior strength of Damascus sabre was due to the presence of carbon nanotubes (CNT). This was the first observation of CNTs in ancient objects which dates back to the sixteenth–eighteenth century.

In this work, we report the evidence of CNTs presence in the black coatings that were used in the inner walls of pottery shards that are excavated from Keeladi, Tamilnadu, India. Radiocarbon dating indicated the Keeladi settlement's period fall in the range of sixth–third century BC^5^. To the author’s knowledge, the discovery of CNT in the Keeladi shards is the oldest among the nanostructure that are reported so far from the ancient artefacts elsewhere.

## Materials and methods

**Figure 1:**
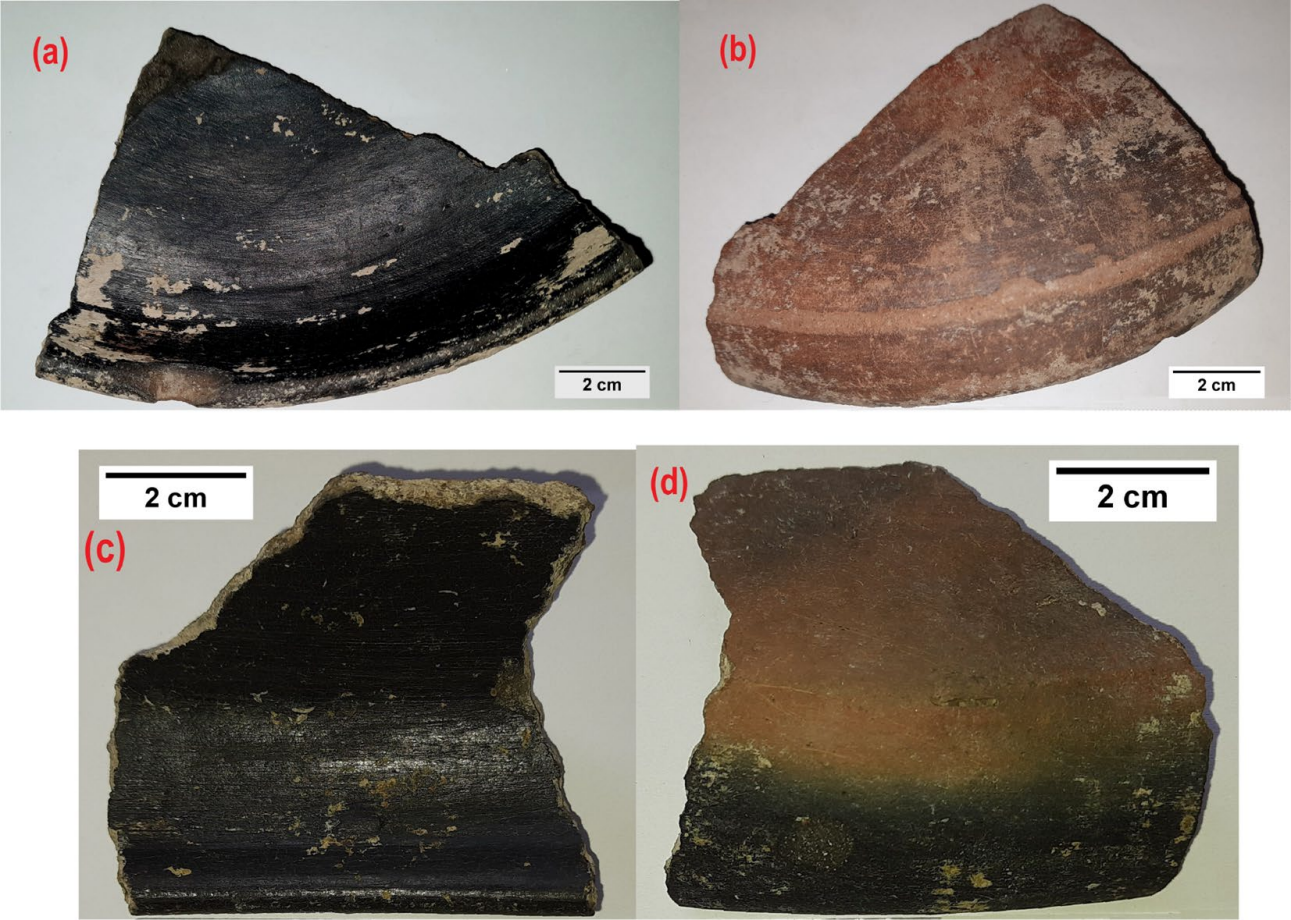
Keeladi Pottery shards (**a**,**c**) Inner portion showing the shining black coating, (**b**,**d**) Outer portion of the pottery shards.

The inner portion of pottery shards excavated from Keeladi had black coating. From visual inspection it appeared shiny, hard and exhibited endurance. Figure 1 shows the images of shreds from Keeladi site.

The black coatings shown in Fig. 1(a,c) were characterized using analytical techniques such as Micro-Raman spectroscopy, X-ray photoelectron spectroscopy (XPS), and by Transmission electron microscopy (TEM). The surface of the black coating was cleaned and Raman measurement was performed for the inner side of the shards as such using LABRAM-HR Confocal laser Micro-Raman spectrometer using 532 nm laser as an excitation source. In a separate analysis the inner black coating was separated, fractionated and drop-casted on a surface as detailed. Inner black coating of the shard up to 1 mm was removed using a surgical knife. The material was grounded on a mortar to fine powder and sonicated in 1 ml of water using probe sonicator. The supension was centrifuged in 50:50 water/glycerol mixture for 10 min at 2500 RPM. The top most layer of the centrifuged solution was collected and washed with acetone by centrifuging the solution at 1000 RPM for 10 min. The process was repeated for three times. Thus obtained suspension was drop casted on a gold coated silicon wafer and micro-Raman measurements were recorded using a BLZE-100 h EMCCD camera (Princeton instruments) equipped with Acton SP2500 monochromator and a confocal microscope. A 532 nm continuous wave, tunable power laser is used for the excitation. All the Raman measurements were carried out at room temperature at lower laser intensity to avoid the sample damage. For TEM, the inner black coating was removed using surgical knife and the resulting black coloured powder was sonicated in acetone and drop casted in carbon free Pd and Cu holey grids and imaged under 200 keV. The images were acquired using FEM-2100 Plus electron microscope with Cu holey grid and by FEI Techani T20 electron microscope with Pd grid. Carbon free grids were specifically chosen to avoid confusions arise upon carbon signature from the samples as reported by Klie et al.^6^, and to prevent from hydrocarbon contaminations^7^. The cleaned samples free from surface contaminants were used for X-ray photoelectron analysis using PHI VersaProbe III with Al Kα x-ray source.

## Results and discussion

Pottery shards, metal pieces and fossil remnants are more common in all archaeological excavations. However, the uniqueness of Keeladi shards is the black coating which remained intact for more than two thousand years. The most interesting factor is the smoothness also preserved with less degradation. Among the shards inspected, some pieces still retaining the smooth shiny surface intact. Since the coating is more than 2000 years old, unless it possesses robust mechanical and chemical stability to resist or isolate from the varying environmental conditions it becomes practically impossible to be stable for such long duration. In order to understand the nature of the coating and its constituents, Raman spectrum was recorded on the inner surface.

**Figure 2:**
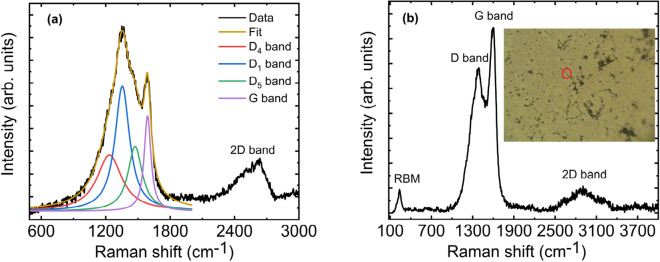
(**a**) Micro-Raman spectrum of the inner black coating showing the presence of D, G and 2D band. The Defect (D) band has been de-convoluted in to various components (D_1_, D_4_ and D_5_) as marked in different colors. The presence of D, G and 2D band confirms the presence of hexagonal graphitic carbon layer and sp^2^ bonded carbon, (**b**) Micro-Raman spectrum from the tube like features (shown in the inset) obtained by drop casting the fractionated suspension on the gold coated silicon surface. The spectrum clearly shows the presence of radial breathing mode (RBM) from the single walled-carbon nanotubes.

The spectrum obtained from the coating confirmed that it is indeed a carbon form as shown in Fig. 2a. It reveals the presence of first-order Raman transition associated with the Defect band (D) and the graphite band (G) of carbon. The D and G band are entangled with few more signatory peaks. The deconvoluted bands of each shoulder peak are shown in different colour code attributing the presence of various components. The band observed at 1589 cm^−1^ corresponds to in-plane vibration (E_2g_ mode) of the sp^2^ bonded carbon, generally indicated as G band. The de-convoluted D band observed at 1354 cm^−1^, 1234 cm^−1^, and 1473 cm^−1^ are corresponds to the defects in the hexagonal graphitic carbon layer, commonly indicated as D_1_, D_4_, and D_5_ band respectively^8^. The broad absorption feature observed around 2600 cm^−1^ is attributed to the second-order Raman transition commonly called as 2D band. The D and G peaks indicate that the black coating is indeed a carbonaceous in nature but not amorphous carbon^9^. It may correspond to disorder graphite or defective graphene oxide or other carbon allotropes. The ratio of intensity of D to G band (I_D_/I_G_) is found to be 1.28. Similarly, the ratio of intensity between 2D and G band (I_2D_/I_G_) is found to be 0.6. The crystallite size in the direction of graphitic plane is calculated by using the formula^10,11^.

1 $$ {\text{L}}_{{\text{a}}} \left( {{\text{nm}}} \right) = \left( {{2}.{4} \times { 1}0^{{ - {1}0}} } \right) \cdot \lambda_{{\text{I}}}^{{4}} \cdot \, \left( {{\text{I}}_{{\text{D}}} /{\text{I}}_{{\text{G}}} } \right)^{{ - {1}}} $$

where L_a_ is the crystallite size in nm, λ_I_ is the wavelength of the excitation laser (532 nm). From the Eq. (1), the crystallite size (L_a_) is calculated to be 15 nm. For pure defect free graphite, the G band occurs at 1575 cm^-110^. Because of the presence of oxygen functional groups, the G band is blue shifted to 1589 cm^−1^^12^. Further, the 2D band around 2600 cm^−1^ is very broad due to the presence of several oxygen functional groups and the spectrum is very similar to graphene oxide^12^. The microscopic image of the fractionated suspension from the inner black coating drop-casted on the surface used for the Raman measurement is shown as an inset in Fig. 2b. The image clearly shows several tubular like structures. Raman spectrum was recorded by pointing the laser on the tubes as shown in the encircled part of inset of Fig. 2b. Spectral features of all the measured spectrum on different tube like structures are identical. This allows us to average the spectrum to increase the signal to noise ratio. The averaged spectrum is shown in Fig. 2b. The spectrum clearly shows the presence of radial breathing mode (RBM) at 236 cm^-1^. The observation of RBM clearly indicates the presence of single-walled carbon nanotubes (SWCNT) as this feature is absent in all other forms of carbon allotropes.

**Figure 3:**
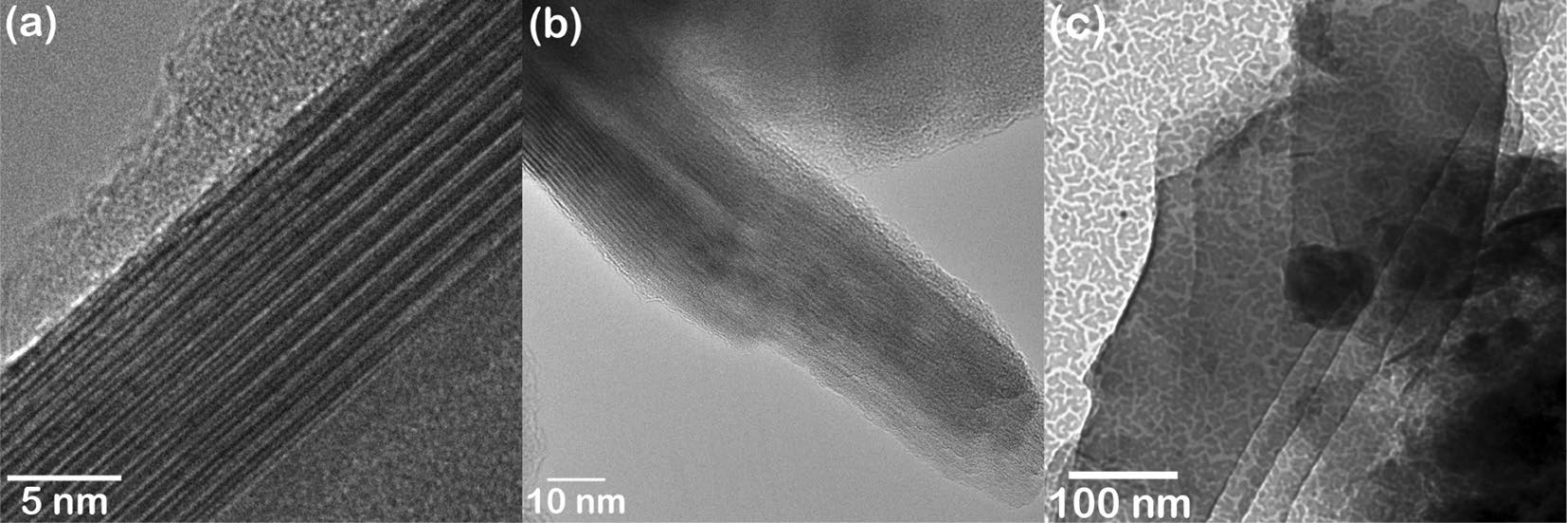
(**a**) SWCNT bundles observed from the coating (smaller spacing is the separation between two walls and larger spacing is the tube inner diameter) (**b**) MWCNT with curling and damages and (**c**) Stack of graphene oxide sheets.

To get insights on the nature of carbon species observed in the Raman spectrum, TEM analysis was carried out to reveal its structure and morphology. Figure 3a shows the presence of bundles of SWCNT. Image in Fig. 3(a,b) were acquired with Cu holey grid using FEM-2100 Plus electron microscope and Fig. 3c was acquired with 2–3 nm Pd coated grid using FEI Techani T20 electron microscope. In order to retain the original signature of the coating, minimal sample preparation has been adapted to realize electron transparent region. As a result, dark contrast was observed in major portion of the grid indicating bulk nature. However, the electron transparent regions revealed interesting features such as randomly distributed SWCNT bundles and MWCNTs.

The average tube diameter was found to be 0.6 ± 0.05 nm and the wall separation was 0.2 ± 0.01 nm (Fig. 3a). The variation in CNT diameter can be identified due to the bending and twisting effect^13^. A close observation of the bundle confirms minuscule bending which might be the cause of fluctuation. It is interesting to note that the smallest theoretical limit for the diameter of CNT is 0.4 nm^14^. Experimentally 0.3 nm was also observed but these small diameter nanotubes are seen as an innermost part of the MWCNT^15^. For SWCNT, the smallest diameter reported was 0.43 nm^16^. It is very surprising to observe SWCNT from an archaeological artefact with 0.6 ± 0.05 nm and has been stable for around 2600 years. Figure 3(b) shows the MWCNT observed from the coating. The spacing between the walls was found to be as 0.34 nm which is in good agreement with the spacing between the (002) lattice plane of the graphite. Also, the inner diameter of the MWCNT is 3 ± 0.15 nm. Curling and damage seen in the tube might be the reason for differences in inner diameter as reported in literatures^13^. Our TEM study confirms the presence of CNTs in the black coating. Although it was not the only constituents, there were regions which revealed the presence of layered sheets as shown in Fig. 3c.

**Figure 4:**
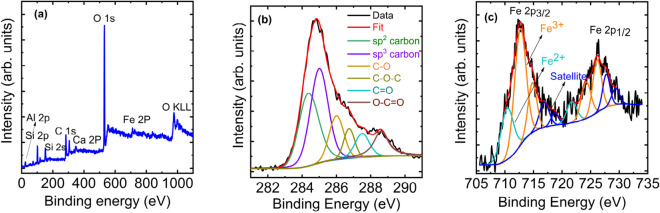
XPS spectrum of the black coating. (**a**) Survey scan showing the presence of various elements, (**b**) Deconvoluted high resolution C1s XPS spectrum, (**c**) Deconvoluted high resolution Fe 2p XPS spectrum. The ratio of Fe 2p_3/2_/Fe2p_1/2_ is 1.97 which is very close to the theoretical estimate as 2.

It is interesting to know the presence of CNTs from the samples that date backs to sixth—third century BC, particularly, given the kind of tools availed at those periods. From the modern synthesis route, we know that elements like Fe, Si and Al can act as a nucleation site for CNTs^17^. The XPS spectra as shown in Fig. 4a indicate the presence of Fe, Al, Si, etc. possibly in the form of their oxides. The XPS data shown in Fig. 4b shows the presence of oxygen functional groups. The (I_D_/I_G_) and (I_2D_/I_D_) values from the Raman spectra are often used as a parameter to estimate the degree of defects that arise by the presence of oxygen atoms in the graphitic plane. Based on the presence of broad 2D band and by low (I_2D_/I_D_) and high (I_D_/I_G_), the sheet found in Fig. 3c is attributed as graphene oxide.

The presence of sp^2^ carbon domains measured from the high-resolution C1s spectrum shown in Fig. 4b confirms the Raman findings^18,19^. High resolution spectra of Fe shown in Fig. 4c indicate that it is present in both + 2 and + 3 oxidation state^20–22^ which possibly aided the CNT formation acting as catalyst.

At this moment, the source of carbon for the coating remains unknown. The C1s x-ray photoelectron spectrum indicates the presence of several functional groups such as carbonyl, ether, carboxyl, and alcohol indicating vegetal source might have been used as a source of carbon during the manufacturing of potteries to form the black coating. Iron observed in the sample might have originated from the vegetal source itself or the soil. So the more scientific possibility would be the plant-based material should have been carbonized, forming different carbon allotropes at high temperature achieved during the firing process of pottery. The presence of iron in the plant source and also the soil might have catalysed the carbon to form SWCNT and MWCNT. High temperature present in the firing process of pottery making might have favoured the formation of observed nano structures.

## Conclusion

Different forms of carbon nanostructures including bundles of single-walled carbon nanotubes, multi-walled carbon nanotubes and sheets like structures probably graphene oxide are observed in the inner black coating of the pottery shards of Keeladi. To the best of our knowledge, it is the oldest nanostructures observed till now. In general CNTs and Graphene are known for its superior mechanical strength than the bulk counterpart^23^. The finding of these two carbon forms in the Keeladi coating raises the following questions. (i) Ancient Keeladi settlement know the importance of these properties and adapted it intentionally? (ii) Given the black coating is observed in the inner portion of the shard, if these potteries were used for edible preparation or preservation then ancient civilization might be aware of the cytotoxic nature of CNT and Graphene/graphene oxide sheets! In spite of other unanswered questions, it is interesting to observe the strong footprints of 1D and 2D carbon-based nanomaterials used about 600 BC ago with diameter closer to theoretical limit and retained its stability for around 2600 years.
